# IMPLEMENT A SELF-ADMINISTERED, SMARTPHONE APP-BASED GAIT ASSESSMENT FOR FUTURE INTERVENTION IN OLDER ADULTS

**DOI:** 10.1093/geroni/igae098.1915

**Published:** 2024-12-31

**Authors:** On-Yee (Amy) Lo, Pei-An Lee, Wanting Yu, Junhong Zhou, Brad Manor

**Affiliations:** Harvard Medical School/Hebrew SeniorLife, Boston, Massachusetts, United States; Harvard Medical School/Hebrew SeniorLife, Boston, Massachusetts, United States; Hebrew SeniorLife, Boston, Massachusetts, United States; Harvard Medical School/Hebrew SeniorLife, Boston, Massachusetts, United States; Harvard Medical School/Hebrew SeniorLife, Boston, Massachusetts, United States

## Abstract

Gait metrics reflect functional capacity and sensitively predict adverse health outcomes. Measuring gait performance – under normal (i.e. walking quietly at a self-selected speed) and “dual-task” conditions (i.e. walking while performing an unrelated cognitive task) – is recommended for assessing and monitoring physical and cognitive functions in older adults. However, there is a significant gap in understanding gait performance outside of lab and clinical settings, yet it is greatly needed. Our team developed a smartphone application (App) to capture gait metrics during normal and dual-task conditions. This App provides users with multimedia instructions, enabling them to self-administer the gait assessment remotely and repeatedly. Acquired data were uploaded to a cloud-based server for offline analyses. Our team has validated and deployed this App within several studies focused on older adults across a wide range of physical and cognitive functional limitations. Results suggested that gait metrics derived from the App were highly correlated with those derived from the GAITRite mat. The test-retest reliability of App-derived gait metrics were high for normal and dual-task walking. Older adults with intact cognition (N=17, MoCA >26) or mild cognitive impairment (MCI, N=16, 19< MoCA≤26) can safely, self-administer this App once a day for 5 consecutive days. Older adults with MCI exhibited significantly higher day-to-day variation in gait variability during dual-task walking (0.30±0.14), compared to cognitively intact older adults (0.19±0.09). Our effort also demonstrated an 80-year-old faller could use this App – with a caregiver’s assistance - to measure gait metrics bi-weekly for up to one year.

